# Supplementary material for: Association between COVID-19 Risk-Mitigation Behaviors and Specific Mental Disorders in Youth
Source: medRxiv. 2022 Mar 5:2022.03.03.22271787. Preprint. [Version 1] doi: 10.1101/2022.03.03.22271787 (PMC8923118; doi:10.1101/2022.03.03.22271787)
Supplement: 1 [file NIHPP2022.03.03.22271787V1-supplement-1.pdf]

medRxiv preprint doi: https://doi.org/10.1101/2022.03.03.22271787; this version posted March 5, 2022. The copyright holder for this preprint (which was not certified by peer review) is the author/funder, who has granted medRxiv a license to display the preprint in perpetuity.
It is made available under a CC-BY 4.0 International license .

Supplement. Sample Characteristics by Study Completion Status

	Sample Characteristics				
	Invited to Participate	Collected and Matched to HBN	Completed Survey	Completed HBN Diagnosis	Total Analytic Sample
**Sex**					
Male	1131 (64%)	645 (62%)	590 (62%)	550 (62%)	514 (62%)
Female	649 (36%)	393 (38%)	364 (38%)	332 (38%)	314 (38%)
**Age (years)**					
5-6	118 (7%)	26 (3%)	25 (3%)	21 (2%)	20 (2%)
7-9	565 (32%)	277 (27%)	252 (26%)	229 (26%)	217 (26%)
10-12	552 (31%)	364 (35%)	340 (36%)	321 (36%)	302 (36%)
13-15	302 (17%)	221 (21%)	200 (21%)	185 (21%)	178 (21%)
16+	243 (14%)	150 (14%)	137 (14%)	126 (14%)	111 (13%)
**Family Structure**					
Single caregiver	156 (9%)	95 (9%)	84 (9%)	82 (9%)	70 (8%)
**SES**[1]					
Low	127 (7%)	79 (8%)	71 (7%)	63 (7%)	58 (7%)
Middle	328 (18%)	193 (19%)	178 (19%)	165 (19%)	153 (18%)
High	1255 (71%)	731 (70%)	677 (71%)	649 (74%)	617 (75%)
**Race**					
Caucasian	945 (53%)	525 (51%)	491 (51%)	471 (53%)	444 (54%)
African American	197 (11%)	127 (12%)	115 (12%)	108 (12%)	99 (12%)
Hispanic	150 (8%)	94 (9%)	82 (9%)	76 (9%)	71 (9%)
Asian	50 (3%)	31 (3%)	29 (3%)	28 (3%)	27 (3%)
Other	319 (18%)	200 (19%)	181 (19%)	166 (19%)	158 (19%)
Unknown	119 (7%)	61 (6%)	56 (6%)	33 (4%)	29 (4%)
**Site**[2]					
Staten Island[2]	653 (37%)	366 (35%)	331 (35%)	308 (35%)	288 (35%)
Midtown	467 (26%)	257 (25%)	235 (25%)	226 (26%)	210 (25%)
Harlem	650 (37%)	406 (39%)	379 (40%)	339 (38%)	322 (39%)

Note: [1]Barratt total score was divided into tertiles: Low (3-24), medium (25-45), high (46-66), and missing Barratt Score is not shown in table (N=70, %=4). [2]Mobile Research Vehicle (MRV) site not shown in table (N=10, %=1). [3]Combined Staten Island site and Staten Island Richmond University Medical Center site.
